# Crystal structure and Hirshfeld surface analysis of a third polymorph of 2,6-di­meth­oxy­benzoic acid

**DOI:** 10.1107/S2056989020014553

**Published:** 2020-11-06

**Authors:** Gustavo Portalone

**Affiliations:** a Chemistry Department, "Sapienza" University of Rome, P. le A. Moro 5, I-00185 Rome, Italy

**Keywords:** crystal structure, benzoic acids, polymorphism, hydrogen bond, 2,6-di­meth­oxy­benzoic acid

## Abstract

A third crystalline form of the title compound, C_9_H_10_O_4_, crystallizing in the centrosymmetric monoclinic space group *P*2_1_/*c*, has been identified during screening for co-crystals. The asymmetric unit comprises a non-planar independent mol­ecule with a synplanar conformation of the OH group. The sterically bulky *o*-meth­oxy substituents force the carb­oxy group to be twisted away from the plane of the benzene ring by 74.10 (6)°. The carb­oxy group exhibits the acidic H atom disordered over two sites between two O atoms. A similar situation has been found for the second tetra­gonal polymorph reported [Portalone (2011 ▸). *Acta Cryst*. E**67**, o3394–o3395], in which mol­ecules with the OH group in a synplanar conformation form dimeric units *via* strong O—H⋯O hydrogen bonds. In contrast, in the first ortho­rhom­bic form reported [Swaminathan *et al.* (1976 ▸). *Acta Cryst*. B**32**, 1897–1900; Bryan & White (1982 ▸). *Acta Cryst*. B**38**, 1014–1016; Portalone (2009 ▸). *Acta Cryst*. E**65**, o327–o328], the mol­ecular components do not form conventional dimeric units, as an anti­planar conformation adopted by the OH group favors the association of mol­ecules in chains stabilized by linear O—H⋯O hydrogen bonds.

## Abstract 2

A third monoclinic polymorph of 2,6-di­meth­oxy­bnzoic acid is reported. The acidic O—H bond of the carboxyl group adopts a synplanar conformation.

## Chemical context   

Until now, two polymorphs are known for 2,6-di­meth­oxy­benzoic acid. Polymorph (Iα) crystallizes in the ortho­rhom­bic space group *P*2_1_2_1_2_1_ with one mol­ecule in the asymmetric unit (Swaminathan *et al.*, 1976 ▸; Bryan & White, 1982 ▸; Portalone, 2009 ▸). As a result of the anti­planar conformation adopted by the OH group, the mol­ecular components are associated in the crystal in chains stabilized by linear O—H⋯O hydrogen bonds. Polymorph (Iβ) crystallizes in the tetra­gonal space group *P*4_1_2_1_2 with one mol­ecule in the asymmetric unit (Portalone, 2011 ▸). In the crystal of the second polymorph, the synplanar conformation of the OH group favours the formation of dimers through O—H⋯O hydrogen bonds. In this article, it is reported the crystal structure of a third polymorph, (Iγ), of 2,6-di­meth­oxy­benzoic acid produced unexpectedly during an attempt to synthesize co-crystals of 5-fluoro­uracil with the title compound.
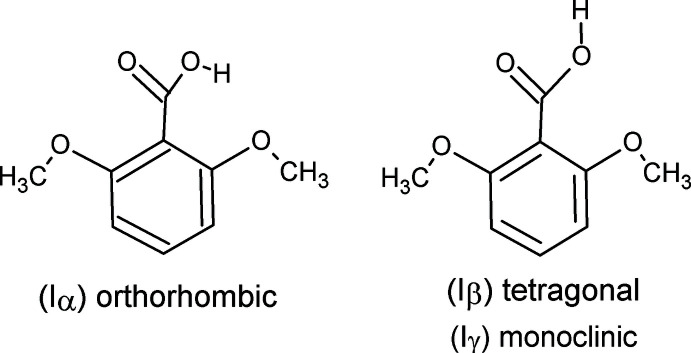


## Structural commentary   

**Figure 1:**
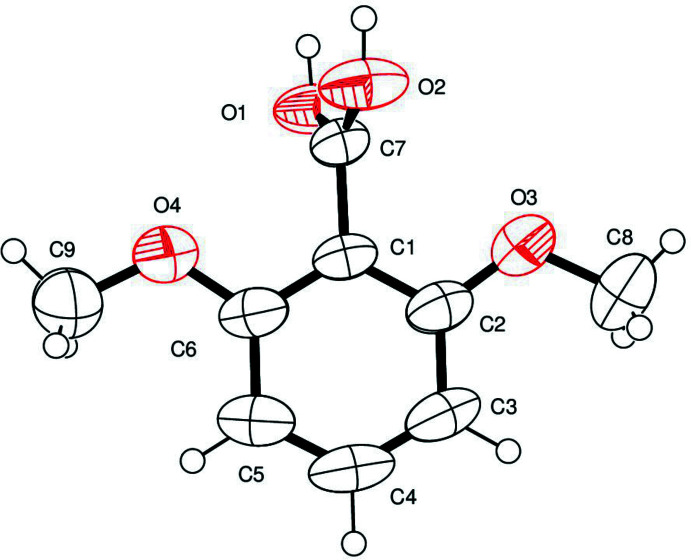
The mol­ecular structure of (Iγ), showing the atom-labeling scheme. Displacement ellipsoids are at the 50% probability level.

The title compound (Iγ) crystallizes in the monoclinic centrosymmetric space group *P*2_1_/*c*, and the asymmetric unit comprises a non-planar independent mol­ecule. The carb­oxy group is twisted away from the plane of the benzene ring by 74.10 (6)° because of a significant steric hindrance of the two *o*-meth­oxy substituents (Fig. 1 ▸). The above angle between the planes is comparable with that found for the ortho­rhom­bic form, 56.12 (9)°, and for the tetra­gonal form, 65.72 (15)°. The carb­oxy group, in which OH adopts a synplanar conformation similar to that observed for the tetra­gonal form, exhibits the carb­oxy H atom disordered over two sites between two O atoms. The pattern of bond lengths and bond angles of the phenyl ring is consistent with that reported in the structure determination of the two previously determined polymorphs, and a comparison of the present results with those obtained for similar benzene derivatives (Colapietro *et al.*, 1984 ▸; Irrera *et al.*, 2012 ▸; Portalone, 2012 ▸) shows no appreciable effects of the crystal environment on the ring deformation induced by substituents.

## Supra­molecular features   

**Figure 2:**
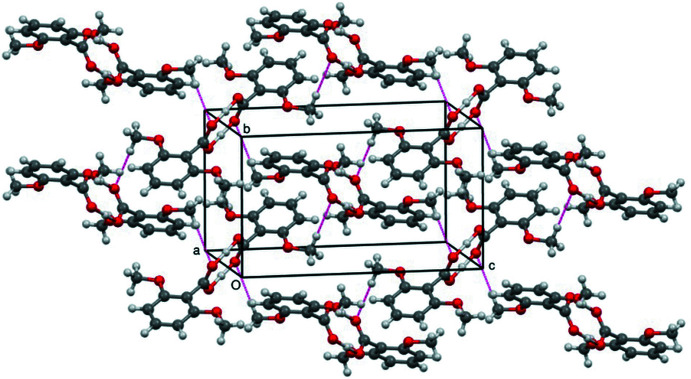
Crystal packing diagram for (Iγ) viewed approximately down the *a* axis. All atoms are shown as small spheres of arbitrary radii. Hydrogen bonding is indicated by red dashed lines.

Analysis of the crystal packing of (Iγ), (Fig. 2 ▸), shows that the mol­ecular components form the conventional dimeric units observed in benzoic acids (Leiserowitz, 1976 ▸; Kanters *et al.*, 1991 ▸; Moorthy *et al.*, 2002 ▸). Indeed, the crystal structure is stabilized by strong inter­molecular O—H⋯O hydrogen bonds, which link inversion-related mol­ecules into homodimers (Table 1 ▸). These homodimers are then joined by weak C—H⋯O inter­molecular inter­actions of graph-set motif 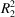
(6) between the meth­oxy and the carb­oxy groups of adjacent mol­ecules to form a two-dimensional network parallel to the *bc* plane.

**Figure 3:**
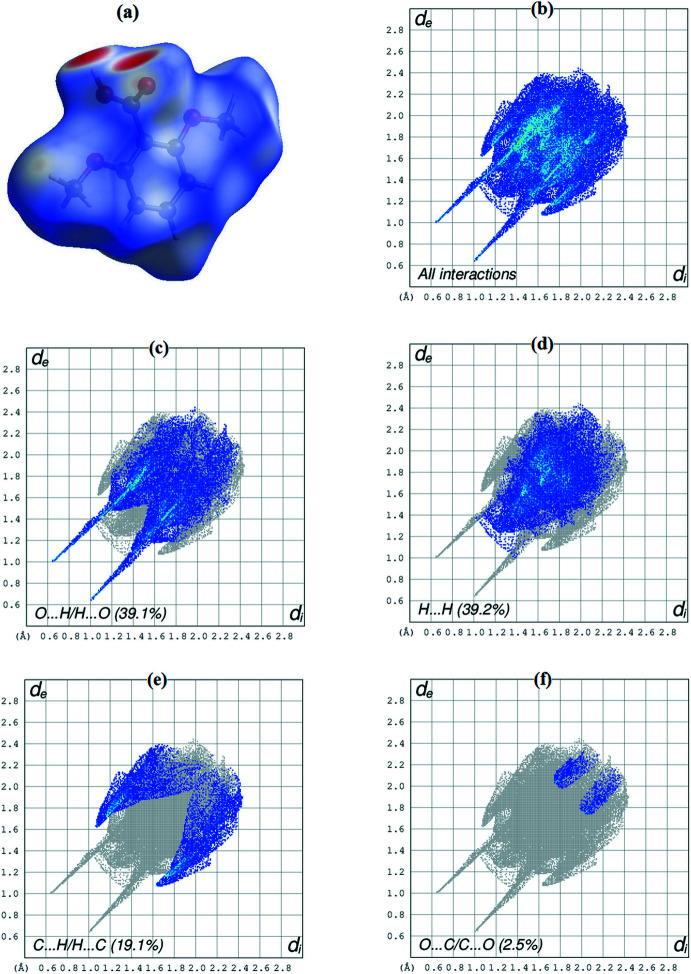
(*a*) A view of the three-dimensional Hirshfeld surface of the title compound mapped over *d*_norm_ with a fixed color scale of −0.742 (red) to 1.283 (blue) a.u. (*b*), (*c*), (*d*), (*e*) and (*f*): decomposed two-dimensional fingerprint plots for the title compound showing various close contacts and their proportional contributions.

The Hirshfeld surface analysis (Spackman & Jayatilaka, 2009 ▸) was carried out using *CrystalExplorer* (Turner *et al.*, 2017 ▸). The surface enables the visualization of inter­molecular contacts over the surface by different colors and color intensity, and shorter and longer contacts are indicated as red and blue spots, respectively. In Fig. 3 ▸ are shown the 3D Hirshfeld surface, modeled by choosing one of the two equally disordered components and mapped over *d*_norm_, and the two-dimensional fingerprint plots, which give the contribution of the inter­atomic contacts to the Hirshfeld surface. The most prominent inter­actions, due to strong O—H⋯O hydrogen bonds, are shown by large and deep red spots on the surface. Small red spots on the surface indicate the areas where close-contact inter­actions due to weak C—H⋯O hydrogen bonds take place. The H⋯H contacts, representing van der Waals inter­actions, and the O⋯H/H⋯O contacts, representing inter­molecular hydrogen bonds, are the most populated contacts and contribute 39.2 and 39.1% of the total inter­molecular contacts, respectively. Other important contacts, such as C⋯H/H⋯C (19.1%), also supplement the overall crystal packing. The contributions of the O⋯C/C⋯O (2.5%) contacts are less significant.

## Database survey   

A search of crystal structure of 2,6-dimeth­oxy benzoic acid alone in the Cambridge Crystallographic Database (CSD version 5.41, May 2020 update; Groom *et al.*, 2016 ▸) yielded four hits as crystalline polymorphs. Three were for the ortho­rhom­bic polymorph: DMOXBA (Swaminathan *et al.*, 1976 ▸), DMOXBA01 (Bryan & White, 1982 ▸) and DMOXBA02 (Portalone, 2009 ▸); the fourth one was for the tetra­gonal polymorph: DMOXBA03 (Portalone, 2011 ▸).

## Synthesis and crystallization   

Polymorph (Iγ) was formed from an unsuccessful co-crystallization between 2,6-di­meth­oxy­benzoic acid and 5-fluoro­uracil. Colorless plate-like crystals were formed by the slow evaporation of an aqueous solution of 2,6-di­meth­oxy­benzoic acid (1 mmol, Sigma Aldrich at 99% purity) and 5-fluoro­uracil (1 mmol, Sigma Aldrich at 99% purity) in a 1:1 molar ratio.

## Refinement   

Crystal data, data collection and structure refinement details are summarized in Table 2 ▸. All H atoms were identified in difference-Fourier maps, but in the refinement all C-bound H atoms were placed in calculated positions, with C—H = 0.97 Å, and refined as riding on their carrier atoms, with *U*_iso_(H) = 1.2*U*_eq_(C_phen­yl_) or 1.5*U*_eq_(C_meth­yl_). A rotating group model was applied to the methyl groups. The remaining two halves of the disordered O-bound H atom, H1 and H2, were refined freely and their *U*_iso_ values were kept equal to 1.2*U*_eq_(O). Site-occupation factors of H1 and H2 refined to 0.53 (3) and 0.47 (3), respectively.

## Supplementary Material

Crystal structure: contains datablock(s) I. DOI: 10.1107/S2056989020014553/is5548sup1.cif

Structure factors: contains datablock(s) I. DOI: 10.1107/S2056989020014553/is5548Isup2.hkl

CCDC reference: 2042162

Additional supporting information:  crystallographic information; 3D view; checkCIF report

## Figures and Tables

**Table 1:** Hydrogen-bond geometry (Å, °)

*D*—H⋯*A*	*D*—H	H⋯*A*	*D*⋯*A*	*D*—H⋯*A*
O1—H1⋯O2^i^	0.86 (6)	1.79 (6)	2.6411 (15)	174 (4)
O2—H2⋯O1^i^	0.81 (6)	1.84 (6)	2.6411 (15)	167 (5)
C9—H9*A*⋯O2^ii^	0.97	2.60	3.571 (3)	178

Symmetry codes: (i) 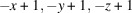; (ii) 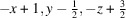.

**Table 2:** Experimental details

Crystal data	
Chemical formula	C_9_H_10_O_4_
*M*_r_	182.17
Crystal system, space group	Monoclinic, *P*2_1_/*c*
Temperature (K)	298
*a*, *b*, *c* (Å)	7.7574 (10), 8.4763 (10), 14.3322 (19)
β (°)	97.526 (12)
*V* (Å^3^)	934.3 (2)
*Z*	4
Radiation type	Mo *K*α
μ (mm^−1^)	0.10
Crystal size (mm)	0.20 × 0.14 × 0.11

Data collection	
Diffractometer	Oxford Diffraction Xcalibur S CCD
Absorption correction	Multi-scan (*CrysAlis RED*; Rigaku OD, 2018 ▸)
*T*_min_, *T*_max_	0.970, 0.999
No. of measured, independent and observed [*I* > 2σ(*I*)] reflections	9067, 2708, 1420
*R*_int_	0.040
(sin θ/λ)_max_ (Å^−1^)	0.704

Refinement	
*R*[*F* ^2^ > 2σ(*F* ^2^)], *wR*(*F* ^2^), *S*	0.049, 0.132, 1.02
No. of reflections	2708
No. of parameters	134
H-atom treatment	H atoms treated by a mixture of independent and constrained refinement
Δρ_max_, Δρ_min_ (e Å^−3^)	0.14, −0.12

Computer programs: *CrysAlis PRO* (Rigaku OD, 2018 ▸), *SIR2004* (Burla *et al.*, 2005 ▸), *SHELXL2014/7* (Sheldrick, 2015 ▸) and *WinGX* (Farrugia, 2012 ▸).

## supplementary crystallographic information

### Crystal data

**Table:**

C_9_H_10_O_4_	*F*(000) = 384
*M**_r_* = 182.17	*D*_x_ = 1.295 Mg m^−^^3^
Monoclinic, *P*2_1_/*c*	Mo *K*α radiation, λ = 0.710689 Å
*a* = 7.7574 (10) Å	Cell parameters from 1806 reflections
*b* = 8.4763 (10) Å	θ = 2.9–32.6°
*c* = 14.3322 (19) Å	µ = 0.10 mm^−^^1^
β = 97.526 (12)°	*T* = 298 K
*V* = 934.3 (2) Å^3^	Tablets, colourless
*Z* = 4	0.20 × 0.14 × 0.11 mm

### Data collection

**Table:**

Oxford Diffraction Xcalibur S CCD diffractometer	2708 independent reflections
Radiation source: Enhance (Mo) X-ray source	1420 reflections with *I* > 2σ(*I*)
Graphite monochromator	*R*_int_ = 0.040
Detector resolution: 16.0696 pixels mm^-1^	θ_max_ = 30.0°, θ_min_ = 2.9°
ω and φ scans	*h* = −10→10
Absorption correction: multi-scan (*CrysAlis RED*; Rigaku OD, 2018)	*k* = −11→6
*T*_min_ = 0.970, *T*_max_ = 0.999	*l* = −19→20
9067 measured reflections	

### Refinement

**Table:**

Refinement on *F*^2^	Hydrogen site location: mixed
Least-squares matrix: full	H atoms treated by a mixture of independent and constrained refinement
*R*[*F*^2^ > 2σ(*F*^2^)] = 0.049	*w* = 1/[σ^2^(*F*_o_^2^) + (0.0439*P*)^2^ + 0.0492*P*] where *P* = (*F*_o_^2^ + 2*F*_c_^2^)/3
*wR*(*F*^2^) = 0.132	(Δ/σ)_max_ < 0.001
*S* = 1.02	Δρ_max_ = 0.14 e Å^−^^3^
2708 reflections	Δρ_min_ = −0.12 e Å^−^^3^
134 parameters	Extinction correction: SHELXL-2014/7 (Sheldrick 2015\bbr000), Fc^*^=kFc[1+0.001xFc^2^λ^3^/sin(2θ)]^-1/4^
0 restraints	Extinction coefficient: 0.031 (4)

### Special details

**Table:**

Geometry. All esds (except the esd in the dihedral angle between two l.s. planes) are estimated using the full covariance matrix. The cell esds are taken into account individually in the estimation of esds in distances, angles and torsion angles; correlations between esds in cell parameters are only used when they are defined by crystal symmetry. An approximate (isotropic) treatment of cell esds is used for estimating esds involving l.s. planes.

### Fractional atomic coordinates and isotropic or equivalent isotropic displacement parameters (Å^2^)

**Table:**

	*x*	*y*	*z*	*U*_iso_*/*U*_eq_	Occ. (<1)
O1	0.50254 (16)	0.32576 (15)	0.56267 (9)	0.0726 (4)	
H1	0.446 (6)	0.372 (5)	0.515 (3)	0.087*	0.53 (3)
O2	0.68711 (16)	0.52450 (15)	0.57631 (9)	0.0776 (5)	
H2	0.643 (7)	0.571 (6)	0.530 (4)	0.093*	0.47 (3)
O3	0.94633 (16)	0.24939 (16)	0.59823 (10)	0.0827 (4)	
O4	0.50328 (18)	0.39851 (17)	0.76761 (9)	0.0900 (5)	
C1	0.73003 (19)	0.31878 (17)	0.68819 (11)	0.0531 (4)	
C2	0.8886 (2)	0.24603 (19)	0.68370 (13)	0.0622 (5)	
C3	0.9743 (2)	0.1717 (2)	0.76309 (15)	0.0767 (6)	
H3	1.0852	0.1199	0.7610	0.092*	
C4	0.8991 (3)	0.1731 (2)	0.84415 (15)	0.0836 (7)	
H4	0.9582	0.1201	0.8992	0.100*	
C5	0.7430 (3)	0.2467 (2)	0.85095 (13)	0.0782 (6)	
H5	0.6940	0.2474	0.9099	0.094*	
C6	0.6574 (2)	0.3199 (2)	0.77134 (12)	0.0648 (5)	
C7	0.63417 (19)	0.39559 (18)	0.60344 (10)	0.0508 (4)	
C8	1.1123 (3)	0.1829 (4)	0.5897 (2)	0.1261 (10)	
H8A	1.2012	0.2385	0.6310	0.158 (12)*	
H8B	1.1358	0.1925	0.5251	0.152 (12)*	
H8C	1.1129	0.0723	0.6072	0.169 (13)*	
C9	0.4198 (3)	0.4098 (3)	0.84841 (17)	0.0986 (7)	
H9A	0.3944	0.3048	0.8698	0.139 (10)*	
H9B	0.3122	0.4684	0.8337	0.129 (9)*	
H9C	0.4950	0.4641	0.8976	0.155 (12)*	

### Atomic displacement parameters (Å^2^)

**Table:**

	*U*^11^	*U*^22^	*U*^33^	*U*^12^	*U*^13^	*U*^23^
O1	0.0660 (8)	0.0702 (8)	0.0718 (8)	−0.0169 (6)	−0.0278 (6)	0.0167 (6)
O2	0.0747 (8)	0.0651 (8)	0.0823 (9)	−0.0180 (6)	−0.0302 (7)	0.0257 (7)
O3	0.0585 (8)	0.1008 (10)	0.0873 (10)	0.0180 (7)	0.0041 (6)	0.0170 (8)
O4	0.0984 (10)	0.1059 (11)	0.0659 (9)	0.0408 (8)	0.0118 (7)	0.0165 (7)
C1	0.0513 (8)	0.0503 (8)	0.0525 (9)	0.0003 (7)	−0.0128 (7)	0.0062 (7)
C2	0.0512 (9)	0.0611 (10)	0.0693 (12)	−0.0009 (7)	−0.0108 (8)	0.0098 (8)
C3	0.0579 (10)	0.0739 (12)	0.0904 (14)	0.0082 (9)	−0.0199 (10)	0.0192 (10)
C4	0.0835 (14)	0.0788 (13)	0.0775 (14)	0.0018 (11)	−0.0309 (11)	0.0234 (10)
C5	0.0925 (15)	0.0788 (12)	0.0580 (11)	0.0040 (11)	−0.0103 (9)	0.0152 (9)
C6	0.0712 (11)	0.0584 (10)	0.0599 (11)	0.0086 (8)	−0.0104 (9)	0.0068 (8)
C7	0.0461 (8)	0.0509 (8)	0.0522 (9)	0.0012 (7)	−0.0057 (6)	0.0047 (7)
C8	0.0704 (16)	0.173 (3)	0.138 (3)	0.0443 (17)	0.0250 (17)	0.037 (2)
C9	0.1151 (19)	0.0953 (17)	0.0891 (16)	0.0249 (15)	0.0273 (15)	0.0135 (14)

### Geometric parameters (Å, º)

**Table:**

O1—C7	1.2563 (17)	C3—C4	1.367 (3)
O1—H1	0.86 (6)	C3—H3	0.9700
O2—C7	1.2472 (18)	C4—C5	1.377 (3)
O2—H2	0.81 (6)	C4—H4	0.9700
O3—C2	1.359 (2)	C5—C6	1.389 (2)
O3—C8	1.425 (2)	C5—H5	0.9700
O4—C6	1.363 (2)	C8—H8A	0.9701
O4—C9	1.402 (2)	C8—H8B	0.9701
C1—C6	1.383 (2)	C8—H8C	0.9701
C1—C2	1.385 (2)	C9—H9A	0.9701
C1—C7	1.4885 (19)	C9—H9B	0.9701
C2—C3	1.391 (2)	C9—H9C	0.9701
			
C7—O1—H1	117 (3)	O4—C6—C1	115.07 (14)
C7—O2—H2	124 (3)	O4—C6—C5	124.95 (19)
C2—O3—C8	118.50 (17)	C1—C6—C5	119.97 (17)
C6—O4—C9	119.91 (16)	O2—C7—O1	123.27 (13)
C6—C1—C2	120.51 (14)	O2—C7—C1	119.20 (13)
C6—C1—C7	118.92 (14)	O1—C7—C1	117.53 (14)
C2—C1—C7	120.57 (16)	O3—C8—H8A	109.5
O3—C2—C1	115.62 (14)	O3—C8—H8B	109.5
O3—C2—C3	124.61 (17)	H8A—C8—H8B	109.5
C1—C2—C3	119.74 (18)	O3—C8—H8C	109.5
C4—C3—C2	118.64 (18)	H8A—C8—H8C	109.5
C4—C3—H3	120.7	H8B—C8—H8C	109.5
C2—C3—H3	120.7	O4—C9—H9A	109.5
C3—C4—C5	122.81 (16)	O4—C9—H9B	109.5
C3—C4—H4	118.6	H9A—C9—H9B	109.5
C5—C4—H4	118.6	O4—C9—H9C	109.5
C4—C5—C6	118.33 (19)	H9A—C9—H9C	109.5
C4—C5—H5	120.8	H9B—C9—H9C	109.5
C6—C5—H5	120.8		
			
C8—O3—C2—C1	177.2 (2)	C9—O4—C6—C5	0.1 (3)
C8—O3—C2—C3	−4.9 (3)	C2—C1—C6—O4	178.43 (14)
C6—C1—C2—O3	178.84 (15)	C7—C1—C6—O4	−2.2 (2)
C7—C1—C2—O3	−0.5 (2)	C2—C1—C6—C5	−0.5 (3)
C6—C1—C2—C3	0.9 (2)	C7—C1—C6—C5	178.92 (15)
C7—C1—C2—C3	−178.51 (14)	C4—C5—C6—O4	−179.32 (17)
O3—C2—C3—C4	−178.01 (17)	C4—C5—C6—C1	−0.5 (3)
C1—C2—C3—C4	−0.2 (3)	C6—C1—C7—O2	106.30 (19)
C2—C3—C4—C5	−0.8 (3)	C2—C1—C7—O2	−74.3 (2)
C3—C4—C5—C6	1.2 (3)	C6—C1—C7—O1	−73.7 (2)
C9—O4—C6—C1	−178.74 (18)	C2—C1—C7—O1	105.66 (18)

### Hydrogen-bond geometry (Å, º)

**Table:**

*D*—H···*A*	*D*—H	H···*A*	*D*···*A*	*D*—H···*A*
O1—H1···O2^i^	0.86 (6)	1.79 (6)	2.6411 (15)	174 (4)
O2—H2···O1^i^	0.81 (6)	1.84 (6)	2.6411 (15)	167 (5)
C9—H9*A*···O2^ii^	0.97	2.60	3.571 (3)	178

Symmetry codes: (i) −*x*+1, −*y*+1, −*z*+1; (ii) −*x*+1, *y*−1/2, −*z*+3/2.
